# Mammalian Sleep Dynamics: How Diverse Features Arise from a Common Physiological Framework

**DOI:** 10.1371/journal.pcbi.1000826

**Published:** 2010-06-24

**Authors:** Andrew J. K. Phillips, Peter A. Robinson, David J. Kedziora, Romesh G. Abeysuriya

**Affiliations:** 1 School of Physics, University of Sydney, Sydney, Australia; 2 Brain Dynamics Center, Westmead Millennium Institute, Sydney Medical School - Western, University of Sydney, Westmead Hospital, Sydney, Australia; 3 Division of Sleep Medicine, Brigham & Women's Hospital, Harvard Medical School, Boston, Massachussetts, United States of America; 4 Center for Integrated Research and Understanding of Sleep, Camperdown, Australia; University College London, United Kingdom

## Abstract

Mammalian sleep varies widely, ranging from frequent napping in rodents to consolidated blocks in primates and unihemispheric sleep in cetaceans. In humans, rats, mice and cats, sleep patterns are orchestrated by homeostatic and circadian drives to the sleep–wake switch, but it is not known whether this system is ubiquitous among mammals. Here, changes of just two parameters in a recent quantitative model of this switch are shown to reproduce typical sleep patterns for 17 species across 7 orders. Furthermore, the parameter variations are found to be consistent with the assumptions that homeostatic production and clearance scale as brain volume and surface area, respectively. Modeling an additional inhibitory connection between sleep-active neuronal populations on opposite sides of the brain generates unihemispheric sleep, providing a testable hypothetical mechanism for this poorly understood phenomenon. Neuromodulation of this connection alone is shown to account for the ability of fur seals to transition between bihemispheric sleep on land and unihemispheric sleep in water. Determining what aspects of mammalian sleep patterns can be explained within a single framework, and are thus universal, is essential to understanding the evolution and function of mammalian sleep. This is the first demonstration of a single model reproducing sleep patterns for multiple different species. These wide-ranging findings suggest that the core physiological mechanisms controlling sleep are common to many mammalian orders, with slight evolutionary modifications accounting for interspecies differences.

## Author Summary

The field of sleep physiology has made huge strides in recent years, uncovering the neurological structures which are critical to sleep regulation. However, given the small number of species studied in such detail in the laboratory, it remains to be seen how universal these mechanisms are across the whole mammalian order. Mammalian sleep is extremely diverse, and the unihemispheric sleep of dolphins is nothing like the rapidly cycling sleep of rodents, or the single daily block of humans. Here, we use a mathematical model to demonstrate that the established sleep physiology can indeed account for the sleep of a wide range of mammals. Furthermore, the model gives insight into why the sleep patterns of different species are so distinct: smaller animals burn energy more rapidly, resulting in more rapid sleep–wake cycling. We also show that mammals that sleep unihemispherically may have a single additional neuronal pathway which prevents sleep-promoting neurons on opposite sides of the hypothalamus from activating simultaneously. These findings suggest that the basic physiology controlling sleep evolved before mammals, and illustrate the functional flexibility of this simple system.

## Introduction

The diversity of mammalian sleep poses a great challenge to those studying the nature and function of sleep. Typical daily sleep durations range from 3 h in horses to 19 h in bats [1], [2], which has led to recent speculation that sleep has no universal function beyond timing environmental interactions, with its character defined purely by ecological adaptations on a species-by-species basis [3]. Consolidated (*monophasic*) sleep, has only been reported in primates [2], whereas the vast majority of mammals sleep *polyphasically*, with sleep fragmented into a series of daily episodes, ranging in average length from just 6 min in rats to 2 h in elephants [1]. Some aquatic mammals (such as dolphins and seals) engage in *unihemispheric* sleep, whereby they sleep with only one brain hemisphere at a time [4]–[6]. This behavior appears to serve several functions, including improved environmental surveillance and sensory processing, and respiratory maintenance [7], although the physiological mechanism is unknown [8], [9]. Determining which aspects of mammalian sleep patterns can be explained within a single framework therefore has important implications in terms of both the evolution and function of sleep. As we show here, although mammalian sleep is remarkably diverse in expression, it is very likely universal in origin.

Recent advances in neurophysiology have revealed the basic mechanisms that control the mammalian sleep cycle [10], [11]. Monoaminergic (MA) brainstem nuclei diffusely project to the cerebrum, promoting wake when they are active [12]. Mutually inhibitory connections between the MA and the sleep-active ventrolateral preoptic area of the hypothalamus (VLPO) result in each group reinforcing its own activity by inhibiting the other and thereby indirectly disinhibiting itself. This forms the basis of the sleep-wake switch, with active MA and suppressed VLPO in wake, and vice versa in sleep [10]. State transitions are effected by *circadian* and *homeostatic* drives, which are afferent to the VLPO [13]. The approximately 24 h periodic circadian drive is entrained by light, and projects from the suprachiasmatic nucleus (SCN) to the VLPO via the dorsomedial hypothalamus (DMH) [14]. The homeostatic drive is a drive to sleep that increases during wake due to accumulation of somnogens, accounting for the observed sleep rebound following sleep deprivation [15]. During sleep, somnogen clearance exceeds production and the homeostatic drive decreases. The exact physiological pathway has yet to be fully elaborated, but some important somnogenic factors have been identified, including adenosine (a metabolic by-product of ATP hydrolysis) [16] and immunomodulatory cytokines [17]. The present work uses a model that does not depend on the precise identity of the somnogen (or somnogens), but may help to elucidate its characteristics.

**Figure 1:**
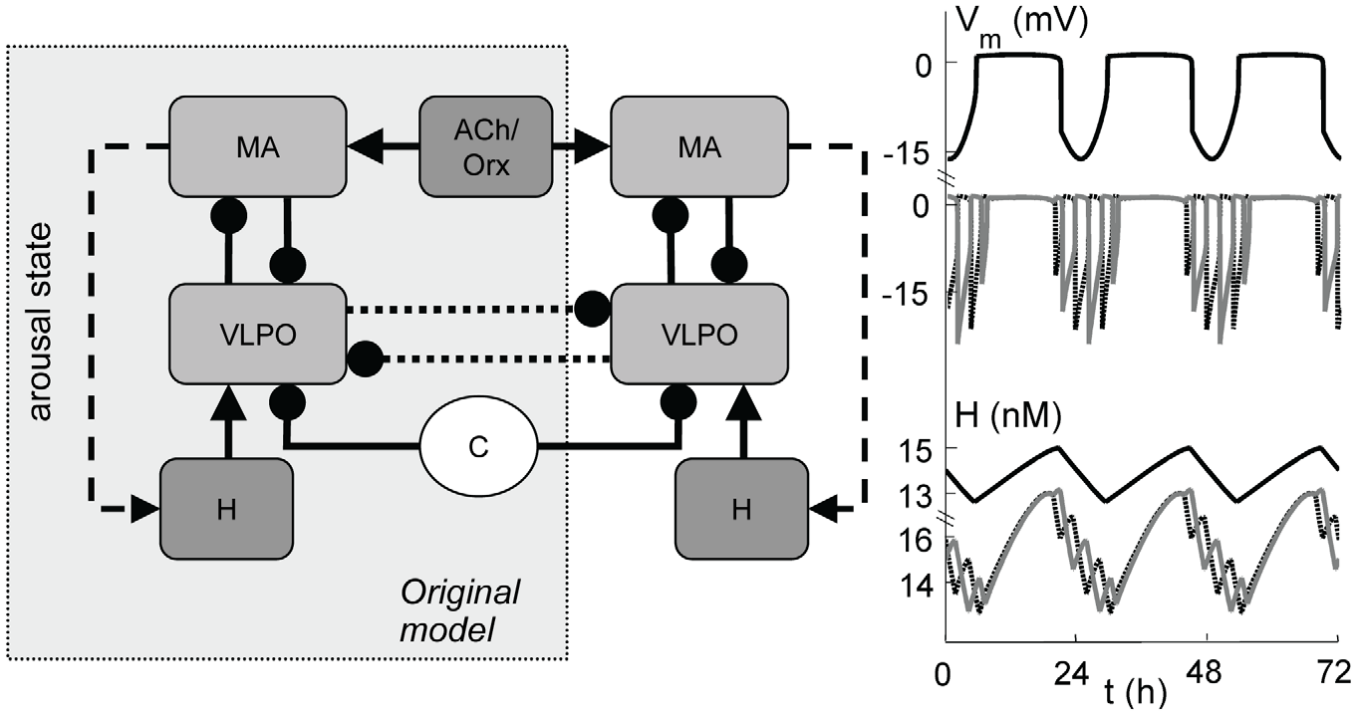
Schematic of the sleep model. Bihemispheric model [18] (gray box), and its extension to model unihemispheric sleep, including MA and VLPO populations, and circadian (C), homeostatic (H), and cholinergic/orexinergic (ACh/Orx) drives. Arousal state feeds back to H. Pointed and rounded arrowheads indicate excitatory and inhibitory connections, respectively. To model unihemispheric sleep we add an inhibitory VLPO-VLPO connection (dotted arrows). Time series are shown alongside MA and H, showing simulated human bihemispheric (top) and dolphin unihemispheric sleep (bottom), with solid and dashed lines distinguishing the hemispheres.

Whether the above system can account for the wide variety of mammalian sleep patterns is unknown. Is the sleep-wake switch a universal physiological structure among mammals? Or are the qualitative differences in sleep-wake patterns between species such as rats and dolphins due to fundamentally different mechanisms? To answer these questions we apply a recent quantitative physiologically-based model [18], [19]; this approach allows the underlying physiological structure to be related to the observed dynamics. As shown in Fig. 1, the model includes the MA and VLPO groups, circadian and homeostatic drives to the VLPO, and cholinergic and orexinergic input to the MA (for mathematical details, see Methods). The model is based on physiological and behavioral studies of a small number of species, including rats, mice, cats, and humans, and has been calibrated previously to reproduce normal human sleep and recovery from sleep deprivation [18], [19]. But as we will show, the model is also capable of reproducing the typical sleeping patterns for a wide range of mammalian species, including both terrestrial and aquatic mammals.

## Results

### Bihemispheric sleep patterns of mammals

With nominal parameter values (given in Methods), the model has previously been shown to reproduce normal human sleep patterns, with approximately 8 h of consolidated sleep, and relatively rapid (approximately 10 min) transitions between wake and sleep [18], as shown in Fig. 1. We found that by varying just two of the model parameters, the model could be made to reproduce the bihemispheric sleep patterns of a wide variety of mammals, including many in which the neuronal circuitry controlling sleep rhythms has not been examined. These parameters were: (i) the homeostatic time constant, determining the rate of somnogen accumulation and clearance, and (ii) the mean drive to the VLPO, provided by the SCN, DMH and other neuronal populations. The homeostatic time constant was found previously to be approximately 45 h for humans, based on the rate of recovery from total sleep deprivation [19], but we found here that reducing it below 16 h resulted in polyphasic sleep, as seen in most other mammals. This is because a shorter time constant causes somnogens to accumulate more quickly during wake, and dissipate more quickly during sleep, resulting in more rapid cycling between wake and sleep. Increasing the mean inhibitory drive to the VLPO was found to decrease daily sleep duration with little effect on the other dynamics.

**Figure 2:**
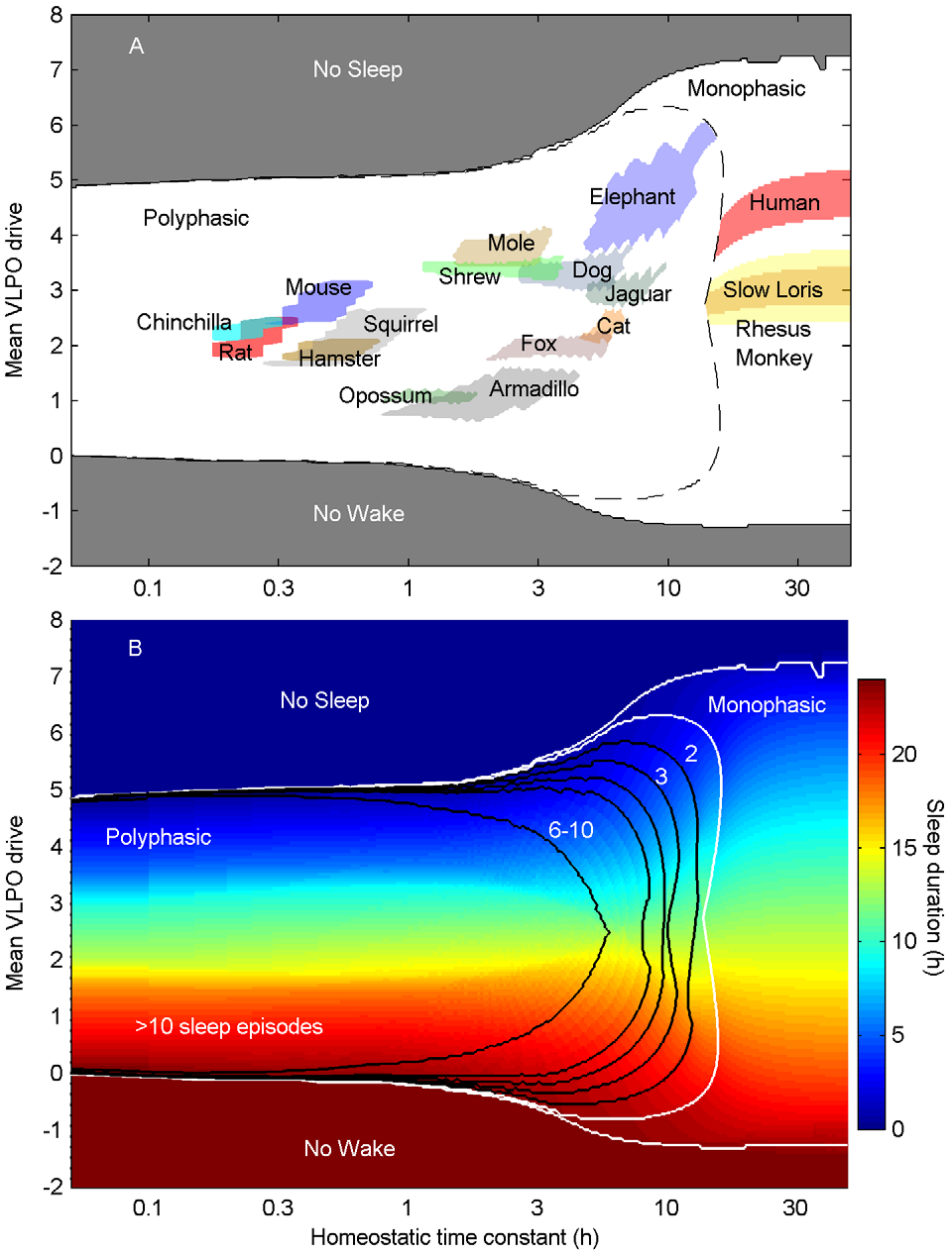
Map of system dynamics corresponding to different mammalian species. (A) Parameters corresponding to sleep patterns of 14 mammalian species, using data from the following sources: rat, mouse, hamster, squirrel and chinchilla [20], eastern mole [21], asian elephant [22], dog [23], jaguar [24], cat [25], fox [26], opossum [27], armadillo [28], common shrew [1], rhesus monkey [29], and slow loris [30]. (B) Sleep duration for these parameters, with zones corresponding to different numbers of sleep episodes per day, as labeled.

Fitting the model to experimental data for 17 species in which both average daily sleep duration and average sleep episode length have been reliably reported yielded the map in Fig. 2, showing which regions of parameter space correspond to the typical sleep patterns of each species. (Note that at least some quantitative sleep data is available for over 60 species, but these two measures have not both been reliably reported in most cases.) This map enables classification of mammals based on sleep patterns, and can be further populated in future when more data becomes available. The regions corresponding to the human, rhesus monkey, and slow loris lie in the monophasic zone, but with different mean VLPO drives. In each case, the lower bound for the homeostatic time constant was determined by the boundary of the monophasic zone. For humans, the upper bound of 72 h was previously determined using sleep deprivation experiments [19]. In the absence of experiments detailing recovery from total sleep deprivation in non-human primates, we used the same upper bound for both the rhesus monkey and the slow loris; more data is required to rigorously constrain the homeostatic time constant for these species.

Animals that sleep relatively little, such as the elephant, were inferred to have high values of mean drive to VLPO, while animals that sleep a lot, such as the opossum and armadillo, were inferred to have low values of mean drive to VLPO. Those that cycle rapidly between wake and sleep, such as rodents, were inferred to have short homeostatic time constants (around 10 min to 1 h), while those with fewer sleep episodes per day, such as the jaguar and elephant were inferred to have longer time constants (around 5 h to 10 h), thus lying closer to the boundary between polyphasic and monophasic sleep. The extreme cases of no wake and no sleep may correspond to brainstem lesions, such as those documented clinically [31], and possibly other states of reduced arousal (e.g., hibernation, torpor, coma), although we did not pursue them here.

**Figure 3:**
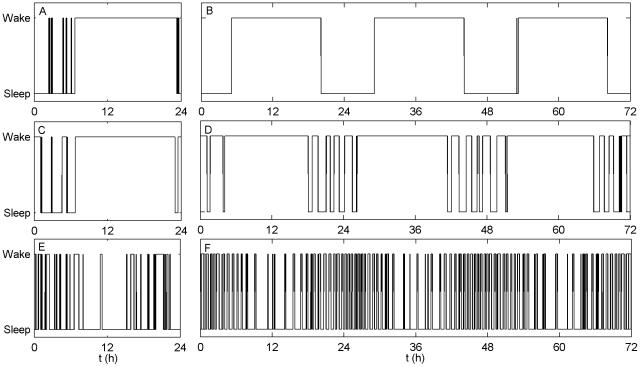
Comparison of experimental data to model output. Time series for wake vs. sleep state are shown for three species, comparing the model to experimental data. Human: (A) data from [32], (B) model (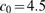, 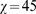 h). Elephant: (C) data from [22], (D) model (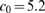, 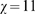 h). Opossum: (E) data from [27], (F) model (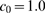, 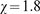 h). Noise is added to the model to make sleep patterns less regular (see Methods for numerical details).

Using parameter values from the appropriate regions in Fig. 2, we generated sample time series for various species. Comparisons to experimental data for the human, elephant and opossum are shown in Fig. 3. In each case, the model reproduced the salient features of the sleep/wake pattern. For the opossum, the circadian signal was shifted in phase by 12 h to reproduce the nocturnal distribution. This is justified by physiological evidence suggesting that temporal niche is determined by how SCN output is modulated by the DMH relay system [11].

### Homeostatic kinetics and brain size

**Figure 4:**
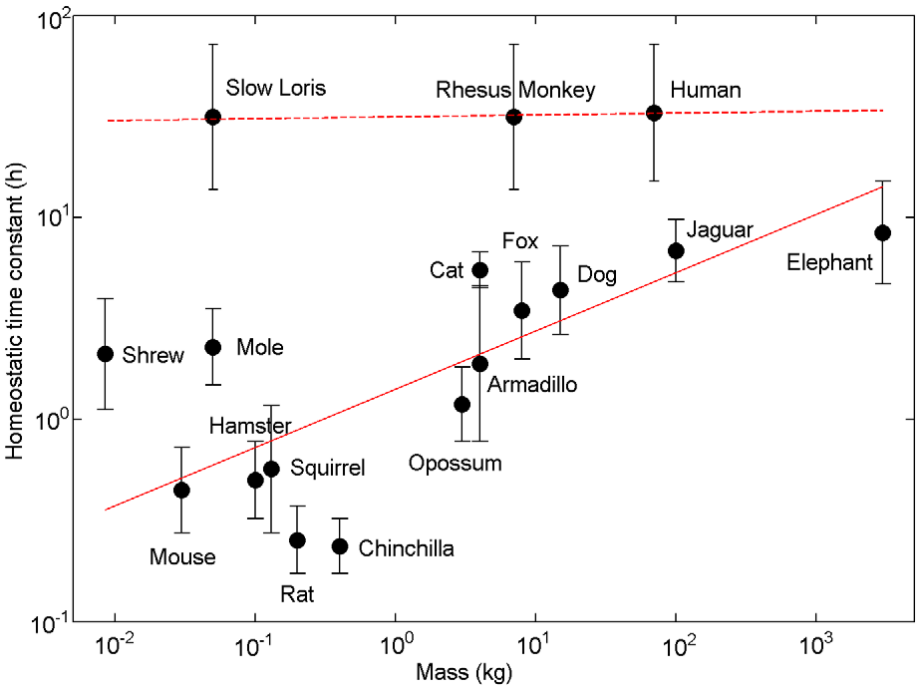
Positive correlation between homeostatic time constant and body mass. Log-log plot of homeostatic time constant (ranges from regions in Fig. 2) vs. body mass for 17 species. Linear fits are shown for non-primates (solid, 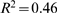), corresponding to a power law with exponent 0.29±0.10 (Mean±S.D. calculated using bootstrapping), and for primates (dashed, 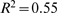) with exponent 0.01±0.26. A linear fit to all species (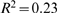) yields an exponent of 0.28±0.12.

Plotting the homeostatic time constants inferred for each species versus body mass in Fig. 4 revealed a positive correlation. Fitting a power-law relationship yielded an exponent of 0.29±0.10 for non-primates. Additional data are required to accurately constrain homeostatic time constants in non-human primates, but using the human-derived upper bound of 72 h yielded an exponent of 0.01±0.26 for primates, and 0.28±0.12 for all species.

Power-law relationships are ubiquitous in biology, although their quantification remains controversial. For mammals it has been found that brain mass 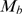 scales as approximately 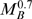, where 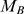 is total body mass, and metabolic power per unit volume scales as 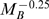 for brain tissue [33]. Without knowing the precise mechanism by which the homeostatic drive is regulated, we nonetheless tested general assumptions that are equally applicable to a wide range of candidate mechanisms. We assumed that somnogen production is proportional to the total power output of the brain (as would plausibly be the case for adenosine), meaning production per unit volume would scale as 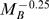, with different production rates in wake and sleep. Furthermore, we made the generic assumption that somnogen clearance rate is proportional to working surface area, where this surface area may be glial, vascular, or otherwise, depending on the exact physiological pathway. The total clearance rate then scaled as 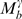, where 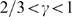, depending on the geometry: 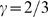 corresponds to surface area scaling as the square of the brain's linear dimension (i.e., as for simple solids), and 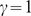 to scaling as its cube (e.g., as for solids with highly convoluted or fractal surfaces). By assuming clearance rate was also proportional to somnogen concentration, the homeostatic time constant was found to be proportional to 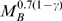 (see Methods for a full derivation). For 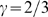, this yielded a power law exponent of 0.23, consistent with that found for non-primates. The smaller exponent found for primates was consistent to within uncertainties with that found for non-primates; more primate data are required to determine whether 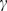 is closer to 1 in primates, or whether both groups follow the same scaling law but with different normalization constants.

### Hypothesized mechanism for unihemispheric sleep

We next turned to modeling unihemispheric sleep by extending the above model to permit distinct dynamics for the two halves of the brain. As shown in Fig. 1, this was achieved by coupling together two identical versions of the original model, each representing one hemisphere. This division in the model was justified by the fact that all nuclei in the VLPO and MA groups are bilaterally paired [12], [34], with the exception of the dorsal raphé nucleus, which lies on the brainstem midline [12]. Separate homeostatic drives were included for each brain hemisphere, based on experimental evidence for localized homeostatic effects in humans, rats and dolphins [35]–[38]. Aquatic mammals that have been observed to sleep unihemispherically spend little or no time in bihemispheric sleep while in water [8] (although fur seals switch to exclusively bihemispheric sleep when on land [39]). Hence, we postulated the existence of a mutually inhibitory connection between the two VLPO groups in aquatic mammals to prevent both activating at once (just as the mutually inhibitory VLPO-MA connection prevents both those groups activating simultaneously), thereby preventing bihemispheric sleep. This connection is presumably absent or very weak in other mammals.

**Figure 5:**
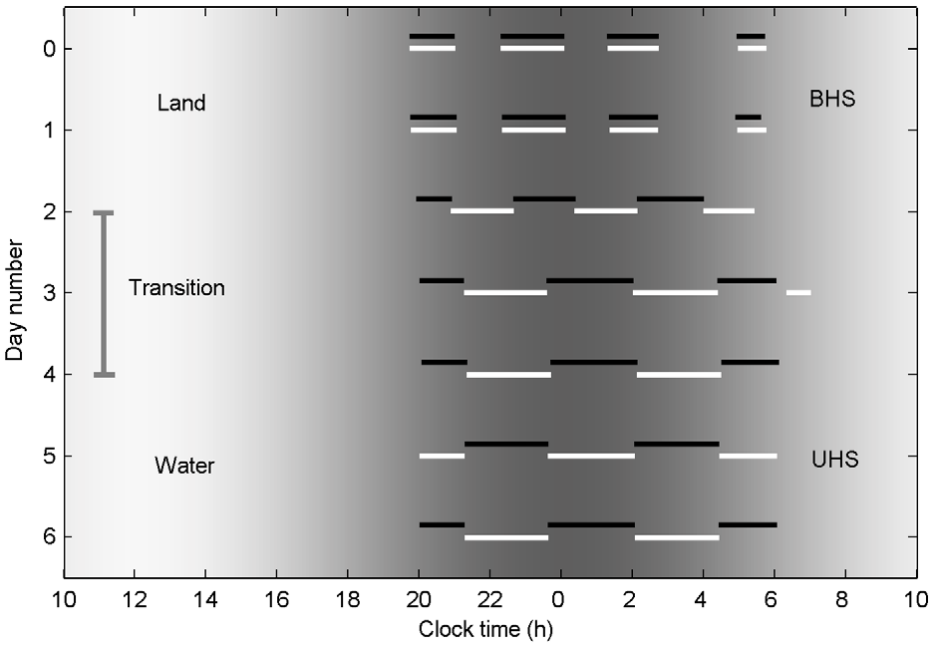
Model simulation of unihemispheric sleep. Simulated transition from polyphasic bihemispheric (BHS) to unihemispheric sleep (UHS), effected by increasing VLPO-VLPO connection strength. Raster plot of sleep for left (white) and right (black) hemispheres, with environmental light level indicated by background brightness. This simulates the behavior of a fur seal in a terrestrial environment on days 0–2 and aquatic thereafter. The VLPO-VLPO connection strength linearly increases from 0 to 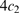 during the transition period on days 2–4.

For VLPO-VLPO connection strengths weaker than a threshold value 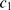 sleep was purely bihemispheric, and above this value at least some unihemispheric sleep episodes occurred. For connection strengths stronger than a higher threshold 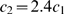 the model exhibited purely unihemispheric sleep, typical of cetaceans. Differing homeostatic pressures between the two hemispheres drove alternating episodes of left and right unihemispheric sleep, with episode length controlled by homeostatic time constant, in a way similar to polyphasic bihemispheric sleep as described above. In Fig. 5, increasing the VLPO-VLPO connection strength was shown to cause a transition from polyphasic bihemispheric sleep to unihemispheric sleep, as for fur seals moving from land to water [6], [39]. Since no other parameter changes were required, we hypothesized that fur seals achieve this readjustment by dynamically neuromodulating the VLPO-VLPO connection strength in response to environmental stimuli. The required strengthening by a factor of somewhat more than 2.4 is reasonable given the magnitudes of typical neuromodulator effects.

## Discussion

We have provided the first demonstration that the neuronal circuitry found in a small number of species in the laboratory, including rats, mice and cats, can account for the sleep patterns of a wide range of mammals. Furthermore, this was achieved by varying only two model parameters, with all others taking fixed values determined previously. The implications of this are far-reaching: universality of this fundamental physiological structure across diverse orders would suggest that its evolution predates mammals. This is consistent with findings that show the monoaminergic system is phylogenetically pre-mammalian [40], and that simple organisms such as the zebrafish share homologous neuronal and genetic control of sleep and wake [41], [42]. Our results also demonstrate the inherent functional flexibility of the sleep-wake switch, which plausibly accounts for its evolutionary success in the face of diverse evolutionary pressures on the sleep-wake cycle. Physiological commonality is also of immense importance when using animals in pharmaceutical development, and for inferring the consequences for humans of animal sleep experiments and genetics.

Our findings suggest that the rate of cycling between wake and sleep is largely determined by the homeostatic time constant, which is inferred to have a positive correlation with body mass. Deviations from this relationship are likely due to selective pressures such as predation, food availability, and latitude. Consistent with this, a previous study found a scaling law of exponent 0.20±0.03 between the characteristic timescale of sleep episode durations (which followed an exponential distribution) and body mass [43]. Mean drive to the VLPO determined sleep duration, and no clear correlation was found between this parameter and body size. Experimental evidence suggests that sleep duration is dictated by interplay between physiological and ecological pressures [44].

The primary advantage conferred by using a physiologically-based model to analyze and interpret data is the ability to relate such behavioral measures to physiology, giving new insights into how interspecies differences in sleep patterns arise. Due to the relative paucity of appropriate data, in this study we made use of all data we could find. This meant combining results of behavioral studies with EEG studies, despite the fact that these methods likely produce slightly different estimates of sleep duration and sleep bout length. While this should not affect our main conclusions, it could fractionally shift the zones in Fig. 2. We thus emphasize the importance of experimentalists continuing to study a wide variety of mammalian species, and encourage them to report metrics such as sleep bout length, total daily sleep duration, and transition frequencies.

While the exact physiological mechanism underlying the homeostatic sleep drive is unknown, some pieces of the puzzle have been identified. Growing evidence points to the role of adenosine accumulation at specific brain sites in promoting sleep. In the rat, basal forebrain adenosine concentration has been found to gradually rise and fall during wake and sleep, respectively, with heightened levels following sleep deprivation [16]. Artificial infusion of adenosine reduces vigilance [45], and the wake-promoting effects of caffeine (which is a competitive antagonist of adenosine) provide additional indirect evidence for adenosine's role in homeostatic sleep regulation. However, the pathway by which adenosine induces sleep is not altogether clear. Adenosine inhibits wake-promoting cholinergic neurons in the basal forebrain, and disinhibits the VLPO via another basal forebrain population [13], [46], yet adenosine agonists continue to promote sleep even after cholinergic neurons are lesioned [47]. Immune signaling molecules such as interleukin-1 (IL-1) and tumor necrosis factor (TNF) have also been linked to homeostatic sleep regulation [17]. Levels of TNF and IL-1 alternate with the sleep/wake cycle, and their exogenous administration induces sleepiness [48]. Furthermore, increased cytokine production during bacterial infection increases sleep duration [48], unless the IL-1 system is antagonized [49]. However, the pathway by which cytokines regulate sleep has yet to be fully elaborated. More critically, no physiological process has been demonstrated to account for the homeostatic drive's timescale, which can be up to a week in the case of chronic sleep deprivation in humans [50]. Adenosine's half life in the blood is only seconds [51], suggesting that clearance and production may be rate-limited further upstream.

In this paper, we assumed that somnogen production and clearance rates are proportional to brain volume and surface area, respectively. The utility of this approach is that it does not require precise knowledge of the physiology underlying the homeostatic drive, because these assumptions are equally valid for a wide range of candidate mechanisms. Using them, we were able to relate scaling laws for metabolism and brain mass to the observed interspecies differences in sleep patterns. Additional data is required to ascertain whether primates follow a different scaling law from non-primates, and if so whether this is due to greater cortical folding, cortical thickness, and neuronal density than most other mammals [52], which could feasibly account for geometrical differences in vascular surface area for instance. Furthermore, additional data is required to determine whether the positive correlation between body mass and homeostatic time constant conforms to a power law. In a similar vein, a theoretical study by Savage and West [53] was able to predict an observed power law relationship between body mass and the ratio of sleep to wake duration, based on the assumption that sleep's primary function is brain maintenance and repair, but the present derivation is the first from a dynamical sleep model.

While sleep/wake patterns are controlled at a fundamental level by systems in the brainstem and hypothalamus, it is worth remembering that sleep is a multi-scale phenomenon, regulated at many levels. For example, synaptic homeostasis may contribute to the local regulation of slow wave activity in the cortex during sleep, and could even play a role in generating the homeostatic drive to the sleep-wake switch [54], [55].

The proposed interhemispheric inhibitory connection in unihemispheric sleepers awaits experimental testing. To date, VLPO afferents have only been studied in animals that sleep bihemispherically, with the great majority of these being ipsilateral [34]. It remains to be seen whether aquatic mammals have a stronger contralateral connection. A question that naturally arises is whether an analogous connection might also be present to some degree in animals that sleep bihemispherically, and whether unihemispheric sleep could be induced by decoupling the hemispheres by other means. Acallosal humans have decreased EEG coherence between hemispheres during sleep, but do not display unihemispheric sleep [56], suggesting that hemispheric synchrony is achieved subcortically. Consistent with this, bisection of the brainstem in cats has been shown to result in all four behavioral states: bihemispheric wake, bihemispheric sleep, and unihemispheric sleep in each hemisphere [57]. This suggests that in bihemispheric sleepers, contralateral excitatory connections between wake-promoting brainstem nuclei and/or the VLPO nuclei may be important to maintaining synchrony. However, bisection of the brainstem in monkeys did not induce unihemispheric sleep [58]. The existence of several other commissures between the hemispheres, including the corpus callosum, may help to explain these results, with one able to compensate for the lack of another in some species. Animals that sleep unihemispherically appear to have evolved multiple physiological changes in parallel to enable this mode of sleep, including a narrow or absent corpus callosum in dolphins and birds, respectively, to reduce interhemispheric coupling [59].

In future, our model could be applied to the sleep of species from other classes, including unihemispheric sleep in reptiles and birds [8]. Furthermore, we could consider explicitly modeling the DMH pathway to explore how temporal niche (diurnal vs. nocturnal vs. crepuscular) is determined. Extending the model to differentiate between REM and NREM sleep could provide additional insights. Using such approaches in parallel with physiological investigations could then help to elucidate the evolutionary development of the sleep-wake switch and its specializations.

## Methods

### Sleep-wake switch model

We begin by reviewing the sleep-wake switch model developed previously; for more details see references [18] and [19]. The model includes the MA and VLPO neuronal populations, and the parameters of the model have been rigorously calibrated by comparison to physiological and experimental data for normal human sleep and recovery from sleep deprivation [18], [19]. Nominal human parameter values are given in Table 1. Each neuronal population has a mean cell-body potential 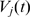 relative to resting and a mean firing rate 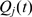, where 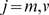 for MA and VLPO, respectively, with
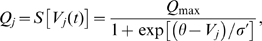
(1)where 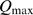 is the maximum possible firing rate, 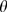 is the mean firing threshold relative to resting, and 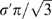 is its standard deviation. Neuronal dynamics are represented by
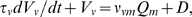
(2)

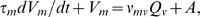
(3)where the 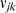 weight the input to population *j* from *k*, 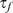 is the decay time for the neuromodulator expressed by group *j*. The orexinergic/cholinergic input 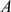 to the MA group is held at a constant average level to smooth out ultradian REM/NREM dynamics [18]. The drive to the VLPO,
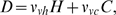
(4)includes homeostatic 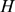 and circadian 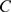 components, where 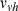 and 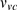 are constants determining the strengths of the homeostatic and circadian drives, respectively. The parameter 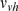 is positive, so that the homeostatic drive promotes sleep; this is consistent with disinhibition of the VLPO by basal forebrain adenosine [13]. The parameter 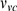 is negative, consistent with the fact that SCN activity promotes wake in diurnal animals [60]. Differences in temporal niche appear to be due in part to an inversion of this signal [60], but as noted in the Discussion, we do not attempt to model this here. The circadian drive is here assumed to be well entrained and so is approximated by a sinusoid with 24 h period,
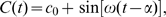
(5)where 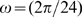 h^−1^, 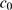 is the mean drive to the VLPO, and 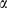 is the initial phase. The homeostatic sleep drive is represented by somnogen concentration 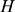, with its dynamics governed by
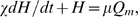
(6)where 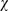 is the homeostatic time constant, and 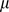 is a constant which determines the rate of homeostatic production. Previously, 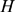 has been considered a model for adenosine concentration in the basal forebrain [18], but this general form is equally applicable to many other candidate somnogens.

**Table 1:** Nominal parameter values for the sleep-wake switch model [19].

Parameter	Value	Unit
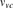	−5.8	mV
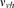	1.0	mV nM^−1^
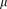	4.4	nM s
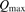	100	s^−1^
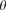	10	mV
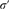	3	mV
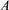	1.3	mV
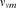	−2.1	mV s
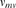	−1.8	mV s
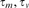	10	s

As shown in earlier work [18], during normal functioning of the model, 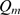 is high (∼5 s^−1^) in wake, 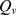 is low (∼0 s^−1^) and 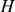 is increasing, while 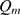 is low in sleep, 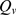 is high and 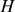 is decreasing. For the purposes of comparing to data, we define the model to be in wake if 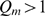 s^−1^, based on comparison with experimental data for MA firing rates [61]. The model differentiates wake vs. sleep states, and we make no attempt to reproduce different sleep intensities or intra-sleep architectures between species.

### Data for calibration

The parameters 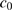 and 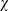 are varied to reproduce mammalian sleep patterns using total daily sleep duration and average sleep episode length as metrics to calibrate against. They have previously been estimated to take the values 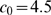 and 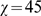 h for humans. These parameters were selected as best able to account for differences in both total daily sleep duration and sleep bout length based on preliminary investigations and previous sensitivity analysis [18]. Data for calibration were derived from an extensive search of the literature to find studies that reported ranges for both metrics, yielding the 17 species used here. Parameter ranges that satisfied these metrics were plotted as the regions shown in Fig. 2. All of the available data were used, with one exception: additional data for non-human primates that sleep monophasically were omitted since we are unable to derive an upper bound for the homeostatic time constant without obtaining data detailing the dynamics of recovery from total sleep deprivation for these species. Those included in the study (the slow loris and the rhesus monkey) are shown for illustrative purposes using the human-derived upper bound of 72 h.

### Incorporating noise

To produce Fig. 3, we add noise terms 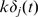 with 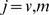 to the right hand sides of Eqs (2) and (3), respectively, so as to make the sleep patterns less regular. The noise 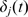 is taken from a normal distribution of mean 0 and standard deviation 1, and 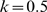 mV h^1/2^/(ΔT)^1/2^, where ΔT is the size of the time step used in the numerical integration. Values of parameters are taken from within the appropriate regions in Fig. 2. For the human, we use 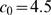, 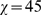 h; for the elephant, we use 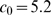, 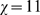 h; for the opossum we use 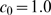, 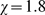 h.

### Unihemispheric sleep

For modeling unihemispheric sleep, the above model, defined by Eqs. (1)–(6) is used identically to model the dynamics of each half of the brain, with the following modification to the VLPO differential equation:
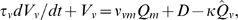
(7)where 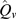 is the firing rate of the VLPO population in the other half of the brain, and 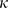 represents the strength of the contralateral inhibitory connection.

### Scaling law

Mammalian brain mass 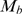 has been found to follow an approximate scaling law
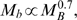
(8)where 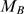 is body mass [33]. Furthermore, the power output of the brain 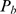 follows,
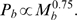
(9)If the total rate of somnogen production in the brain is assumed to be proportional to the total power output of the brain 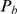, then the rate of somnogen production per unit volume, denoted by 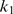, is
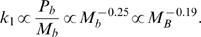
(10)


We assume that the total clearance rate is proportional to the working surface area, which may be glial, vascular, or otherwise. The working surface area will thus scale as the brain's mass, 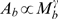, where 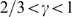 depending on the brain's geometry. Therefore, the rate of somnogen clearance per unit volume, denoted by 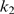, is
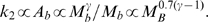
(11)Now, if 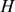 is produced at a rate 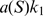 where 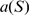 is a factor that depends on the state of arousal 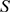 (i.e., production is expected to be higher in wake than in sleep), and 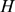 is cleared at a rate 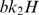, where 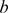 is constant, then
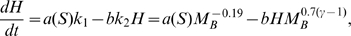
(12)which can be rewritten as
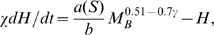
(13)where the homeostatic time constant is 
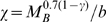
, and 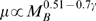. For 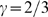, this yields 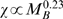 and 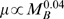, justifying the approximation of holding 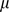 constant while varying 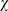 throughout this study.
